# Moving toward universal health coverage with a national health insurance program: A scoping review and narrative synthesis of experiences in eleven low- and lower-middle income countries

**DOI:** 10.1371/journal.pgph.0003651

**Published:** 2025-01-09

**Authors:** Marine Flourence, Eva Jarawan, Mara Boiangiu, Fatima El Kadiri El Yamani

**Affiliations:** 1 Department of Global Health, Georgetown University, Washington, District of Columbia, United States of America; 2 Health, Nutrition & Population Global Practice, The World Bank Group, Washington, District of Columbia, United States of America; 3 Department of Global Health, School of Health, Georgetown University, Washington, District of Columbia, United States of America; Chulalongkorn University College of Public Health Sciences, THAILAND

## Abstract

Universal Health Coverage (UHC) aims to provide access to quality health services to all while avoiding financial hardship. Strategies can include establishing a national health insurance scheme (NHIS). However, variations in the progress exist among countries with an NHIS. This study assesses strategies adopted in low- and lower-middle-income countries (LLMICs) with an NHIS to expand UHC. The research entailed a descriptive, qualitative review of the literature on LLMICs that have implemented an NHIS. PRISMA guidelines were used to identify studies and reports. A total of 569 texts were identified from 4 databases. A total of 78 texts were included, spanning 7 countries from Sub-Saharan Africa and 4 from Asia. The search was conducted in March 2023 and updated in April 2024. An analytical framework was used to systematically collect, analyze, and synthesize key features to review healthcare financing mechanisms and coverage dimensions. Countries generate revenue through various public and private means, including taxes, premiums, and out-of-pocket payments. Some have consolidated revenue streams into a single pool for efficiency, while others maintain separate pools. Healthcare services are procured from public and private providers, differing by country. Fee-for-service is the prevalent payment method, but capitation systems have been attempted to control expenses. Population coverage depends on whether enrollment in an NHIS is mandatory or voluntary and on its enforcement. Service provision can be comprehensive and universal or can vary with specific schemes. Mechanisms to avoid financial hardship can involve premium exemptions or subsidies. Progressing toward UHC requires addressing issues of financial sustainability, cost-containment, enrollment expansion, financial protection, and health equity. While policy options are context-specific, this review showcased experiences for other LLMICs committed to UHC with an NHIS. Recommendations on health financing include increasing the allocation of tax revenues to the insurance scheme, merging risk pools, and adopting strategic purchasing.

## 1. Introduction

Universal Health Coverage (UHC) is defined by the World Health Organization (WHO) as the condition where “all individuals and communities receive the health services they need, without suffering financial hardship.” Achieving UHC is an ambitious goal and is one of the critical targets (Sustainable Development Goals SDG 3.8) the nations of the world set when adopting the SDGs in 2015 [1].

UHC encompasses a comprehensive range of vital health services, from health promotion to prevention, treatment, rehabilitation, and palliative care throughout an individual’s life. UHC not only focuses on the array of services covered but also on their funding, management, and delivery. Consequently, robust, effective, and equitable health systems are essential to support the communities they serve [2].

WHO identifies three key aspects to evaluate a nation’s advancement toward UHC: coverage of the population (identifying who is covered), scope of services (determining which benefits are provided and covered), and financial protection (establishing the extent of costs covered for health service) [2,3].

Many countries have committed to moving towards UHC. For example, this is the case in the Democratic Republic of Congo, which made health a national priority [4,5]. Strategies to progress toward UHC can include the establishment of a national health insurance scheme (NHIS) [6,7]. For example, to remove barriers to healthcare and reduce the financial burden of households, Ghana operationalized its NHIS in 2005 [8]. Rwanda introduced the “*Mutuelles de santé*” in 1999–2000, building on the cultural context of community solidarity. The now-named *Community-Based Health Insurance* (CBHI) was integrated into a NHI system by law in 2015 [6,9]. As a result of the implementation of their national health insurance scheme/system, both Ghana and Rwanda saw an increase in their population coverage. Ghana’s coverage has fluctuated around 35 and 41 per cent between 2011 and 2017 [10]. Rwanda is the only country in Sub-Saharan Africa to have achieved enrollment rates of over 80 per cent, with 83 per cent of its population, both women and men aged 15 to 49, covered by health insurance in 2019 [11,12].

While NHI schemes in low- and lower-middle-income countries (LLMICs) can be a strategy for advancing toward UHC, countries show uneven progress in achieving this goal [1]. What are the UHC approaches and strategies adopted in LLMICs that have implemented an NHIS? To the best of our knowledge, there is no literature that has focused on such strategies. Based on that, we reviewed and identified relevant experiences in different contexts. For this review, a national health insurance scheme refers to any program that requires the payment of a premium by or for each person covered. The health insurance scheme might be community-based, social-based (i.e. wage-based deductions), or private. The insurance scheme offers benefits by covering certain health services. Such insurance schemes may be designed for specific groups (targeted) or the entire population (universal), and participation can be either mandatory or optional (voluntary scheme).

This paper reviews two main themes: i) the health financing mechanisms of selected countries with a national health insurance: what mechanisms countries use to mobilize resources, pool funds, and purchase services, and ii) the coverage dimensions, including the breadth and depth of coverage, and the financial protection mechanisms of these countries. More particularly, we focus on the approaches followed by these countries to expand coverage, the benefits package, and the protection of the most vulnerable.

## 2. Methods

The research entailed a descriptive, qualitative review of the existing literature on LLMICs that have implemented a national health insurance to move toward UHC. For this purpose, we used a scoping review approach. As the main purpose of this study was to gather experiences and outcomes to draw lessons, a scoping review was deemed appropriate for this research to identify key characteristics related to the expansion of UHC [13]. The PRISMA-ScR checklist for the study can be found in the S1 Table.

### 2.1 Literature search strategy

The literature of interest was focused on implemented NHI and key health system features such as healthcare financing and coverage in LLMICs. The terms used for conducting the search are reported in Table 1. All countries classified as LLMICs by the World Bank [14] were included in the literature search.

**Table 1:** Search terms.

Concept 1	Concept 2	Concept 3
National health insurance Social health insurance Universal health coverage	Governance Design Organization Pooling Funding Purchasing Collection mechanisms Revenue Provider payment Benefits package Healthcare financing Financial protection Subsidization Financial sustainability	Low-income and lower-middle-income countries Developing countries

Using the key terms above related to the research topic, a review of the literature was conducted in 4 major electronic databases: PubMed, Embase, Web of Science, and Academic Search Premier. Additional searches for grey literature were conducted intentionally using the same search terms in the official websites of Governments (for example, the Ghana Department of Health and NHIS websites, the website of the Kenya Ministry of Health, etc.) or international development organizations (such as the World Health Organization or the World Bank) (see S1 Fig). All searches were conducted in English. Searches were limited from 2009 to 2024 to ensure that the information was relatively recent and relevant. The initial search was conducted in March 2023 and updated on April 11, 2024. As a result of the literature update, one new study was identified as meeting the inclusion criteria.

All search records were uploaded in EndNote^TM^ X20.6. EndNote was used to eliminate duplicate records. Records were grouped by country.

The articles were independently screened by two reviewers (MF and MB). They were first screened through titles and abstracts to assess their relevance. Only articles that discussed a combination of key concepts, including governance, health insurance, health financing strategies, and health coverage were included for a full-text review.

Articles were excluded if they met one of the following criteria: 1) the studies were conducted in countries where no national health insurance program is running, and 2) the studies were published before the 2009 cut-off date.

### 2.2 Data collection process

A common analytical framework reflecting the key features of health systems in each country was used for data collection. The conceptual framework included concepts around health systems’ characteristics: governance, health financing, and coverage dimensions. An Excel template was developed to systematically extract data from the selected full-text articles. The collected information included 1) characteristics of the study, such as the name of the first author, publication year, name of the country where the study was conducted, study design, and analysis methods; and 2) components of national health systems, including A) governance system; B) healthcare financing features such as funding sources or revenue collection mechanisms, operational pooling structure, purchasing function, and provider payment systems adopted; and C) coverage dimensions such as enrollment in NHI schemes, provision of the benefits package, subsidies to the most vulnerable, and financial protection.

### 2.3 Critical appraisal

Due to the lack of consensus on which tools to assess the methodological quality of non-randomized studies [15], and to the variation of existing literature for the countries included, the researchers did not perform a critical appraisal of the studies identified for this review in order to gather as much evidence as possible.

### 2.4 Data analysis and synthesis

Studies were analyzed and synthesized based on the conceptual framework that included: the distinct functions of health financing, the coverage dimensions, and the factors contributing to progress toward UHC.

## 3. Results and discussion

### 3.1 Characteristics of included articles and of included countries

A total of 78 journal articles and reports were identified for this review (68 journal articles, and 10 reports). Those included for this review span a total of 11 LLMICs (7 from Sub-Saharan Africa, and 4 from Asia).

Two of the countries reviewed are considered low-income (Rwanda and Zambia), while the rest of the countries are lower-middle-income as per the World Bank Classification [14]. Additional social and economic indicators of the countries reviewed can be found in Table 2 [16,17].

**Table 2:** Key indicators of the countries reviewed.

*Country*	*Country by Income*	*Population (2021) million*	*GDP per capita (2021) USD*	*GDP per capita growth (annual %) (2021)*	*CHE % of GDP (2021)*	*GGHE-D as % CHE (2021*	*Human Capital Index (scale 0–1) (2020)*
Ghana	LMI	32.83	2363.3	3.26	4.15	54.06	0.45
Kenya	LMI	53	2081.8	5.45	4.55	48.73	0.55
Nigeria	LMI	213.4	2065.7	3.6	4.08	13.27	0.4
Rwanda	LI	13.46	822.3	8.3	7.32	40.82	0.38
Senegal	LMI	16.87	1636.9	6.1	4.35	25.86	0.4
Tanzania	LMI	63.59	1099.3	1.2	3.36	27.18	0.39
Zambia	LI	19.47	1137.3	1.7	6.62	42.52	0.4
Cambodia	LMI	16.59	1625.2	1.8	7.53	26.58	0.5
Indonesia	LMI	273.8	4332.7	2.97	3.71	59.41	0.54
Philippines	LMI	113.88	3460.5	4.1	5.87	39.33	0.52
Vietnam	LMI	97.47	3756.5	1.7	4.59	42.72	0.69

Abbreviations: CHE: Current Health Expenditure; GDP: Gross Domestic Product; GGHE-D: Domestic General Government Health Expenditure; LI: Low-Income; LMI: Lower-Middle Income.

In terms of health indicators, life expectancy at birth for the countries reviewed ranges between 53 and 74.4 years (for Nigeria and Vietnam, respectively). The fertility rate ranges between 1.9 and 4.8 births per woman (for Vietnam and Tanzania, respectively). Infant mortality rate ranges between 16.9 and 72 per 1,000 live births (for Vietnam and Nigeria, respectively). The maternal mortality ratio ranges between 184 and 917 per 100,000 live births (for Cambodia and Nigeria, respectively) [16]. Finally, all the countries reviewed have a form of National Health Insurance and some have different schemes implemented. Table 3 summarizes key health indicators of the countries reviewed as well as their national health insurance status.

**Table 3:** Health indicators and status of national health insurance.

*Country*	*Life Expectancy at birth*, *total (2020)*	*Fertility rate*, *total (births per woman) (2020)*	*Infant Mortality Rate (per 1*,*000 live births) (2020)*	*Maternal Mortality Ratio (per 100*,*000 live births) (year)*	*National Health Insurance System Implemented*	*Name of National Health Insurance Scheme*
Ghana	64.11	3.6	33	334 *(2017)*	Yes	National Health Insurance Scheme (NHIS)
Kenya	62.68	3.5	31.2	377 *(2014)*	Yes	National Hospital Insurance Fund (NHIF)
Nigeria	53	5.3	72	917 *(2017)*	Yes	National Health
Rwanda	66.78	3.9	30.3	297 *(2015)*	Yes	Social Health Insurance (SHI)
Senegal	68	4.5	29	440 (*2018*)	Yes	Couverture Médicale Universelle (CMU)
Tanzania	66.41	4.8	34.9	642 *(2016)*	Yes	National Health Insurance Fund
Zambia	62	4.4	40	286 (*2013*)	Yes	National Health Insurance (NHI)
Cambodia	70	2.4	21	184 (*2014*)	Yes	National Social Security Fund (NSSF)
Indonesia	68.81	2.19	19.5	282 *(2012)*	Yes	Jaminan Kesehatan Nasional (JKN)
Philippines	72.12	2.8	20.9	206 *(2012)*	Yes	National Health Security Program (formerly named PhilHealth)
Vietnam	75.38	1.9	16.7	No data	Yes	Social Health Insurance (SHI)

### 3.2 Health financing characteristics

**Fig 1:**
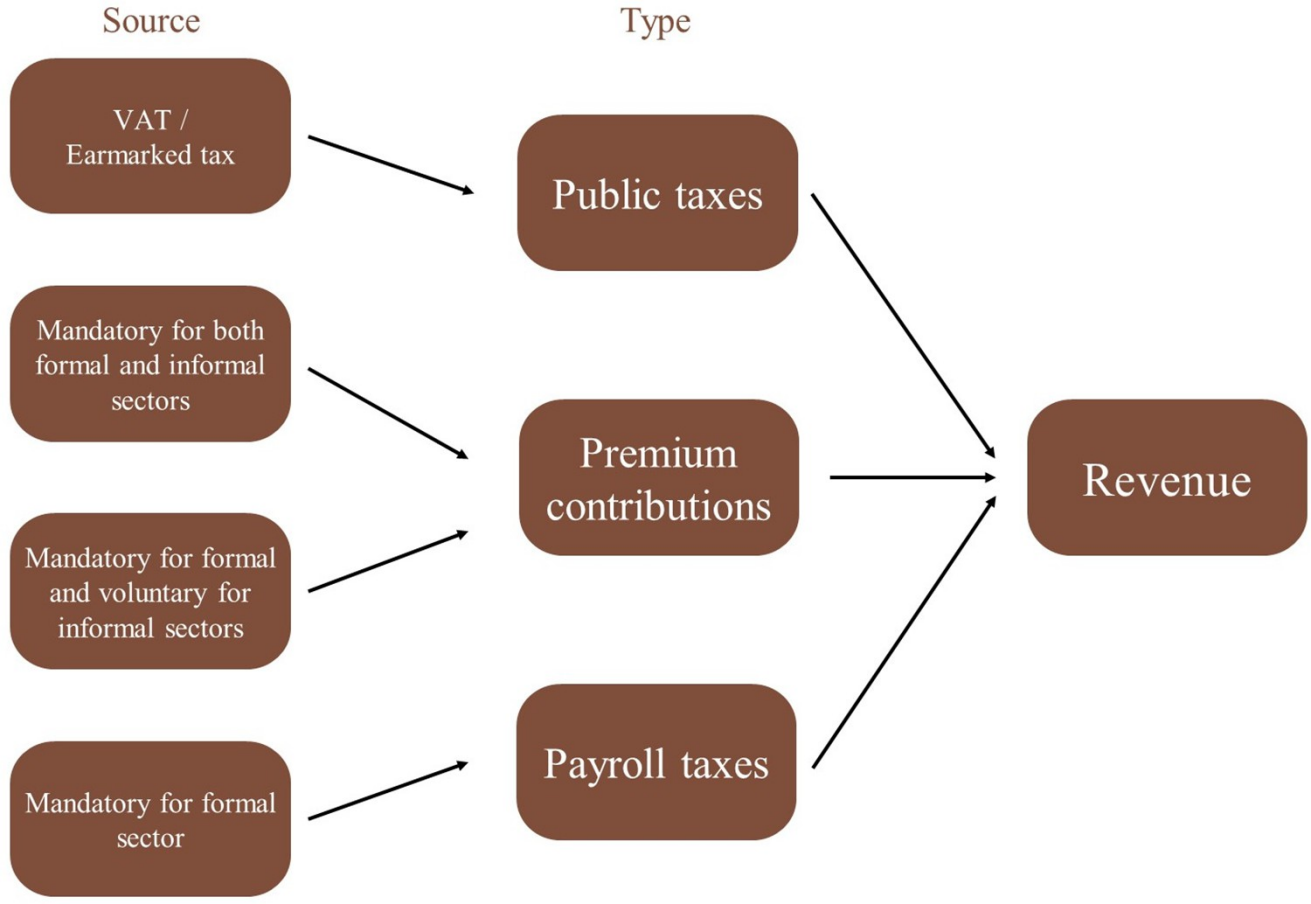
Sources of revenues in countries assessed.

**Fig 2:**
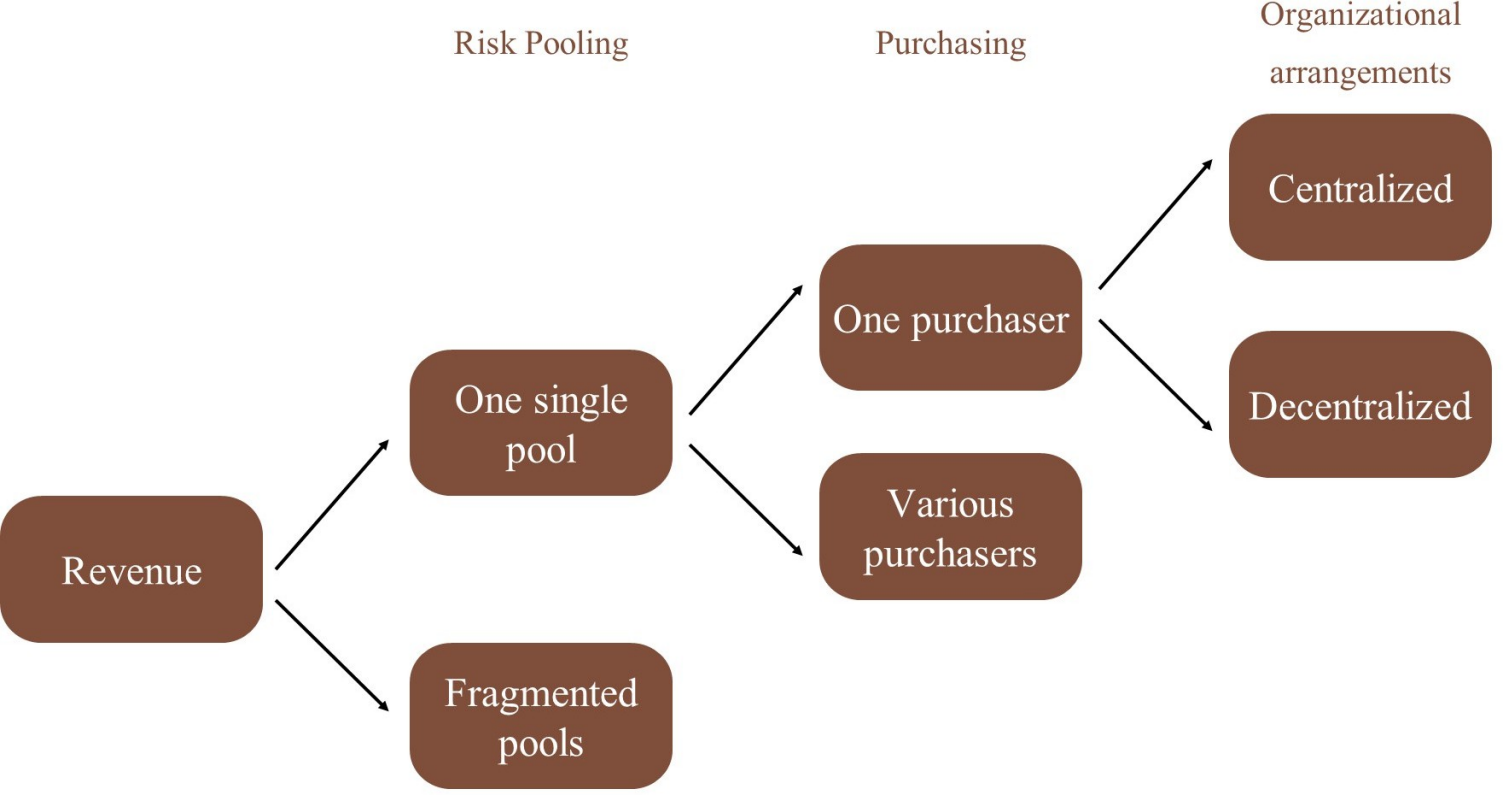
Risk pooling, purchasing, and organizational arrangements of countries assessed.

This section reviews some mechanisms of health financing for the countries reviewed, identifies challenges, and discusses options. Fig 1 shows the scenarios for revenue collection for the countries reviewed. Fig 2 shows arrangements for risk pooling, purchasing, and organizational arrangements.

- Revenue collection

Countries reviewed raise revenues through a combination of public and private sources (general taxes, earmarked taxes, payroll taxes, premium contributions, and out-of-pocket payments) and international assistance (budget support, health programs, etc.). For example, Ghana increasingly relies on tax revenues to fund coverage, and premium contributions account for 3 per cent of the NHIS fund [18]. In Indonesia and Vietnam, revenue sources include governmental contributions from payroll, income, and tobacco taxes [19,20]. In the Philippines, PhilHealth’s funding mainly comes from the premium paid by individuals who are employed, self-employed, or sponsored. For those employed, the premium is capped at 3 per cent of the monthly salary, divided equally between the employee and employer. Self-paying and sponsored members contribute 300 Philippine pesos (PHP) per quarter (US$ 6). The premiums of sponsored members are also covered by local and national government subsidies [21]. Rwanda relies on mandatory premium contributions for both the formal and informal sectors, building on the country’s legislation making contributions from the informal sector compulsory [9]. In contrast, premium contributions are voluntary in the informal sector in Kenya and Tanzania, leading to limited collection of revenues from this source [22,23].

Progressing toward UHC requires addressing long-term financial sustainability considerations. The voluntary nature of premium contributions or the exemptions of contributions may jeopardize such sustainability. While all residents in Ghana, including non-citizens, are eligible for NHIS coverage, not all enrollees are required to pay premiums. Two-thirds of the NHIS enrollees, such as contributors to the Social Security and National Insurance Trust and the most vulnerable, are exempt from premium contributions, which questions the financial sustainability of the scheme that relies primarily on VAT revenues [8,10,18].

Some countries diversify their sources of revenue to fund healthcare expenses. Examples include increasing the allocation of tax revenues to the insurance scheme or enhancing revenue collection through economic growth and improved taxation methods [24,25]. This is the case in the Philippines, where a “Sin Tax” on alcohol and tobacco raised more than 1.2 billion US dollars in its first year of introduction [26].

- Risk-pooling

Several of the countries reviewed have unified their fragmented schemes to create one single pool of revenue, improving equity and efficiency. This is the case of the Rwandan Social Security Board (RSSB), the Ghanaian National Health Insurance Scheme (NHIS), and the Jaminan Kesehatan Nasional (JKN) Insurance scheme in Indonesia.

Since 2014 in Rwanda, the RSSB has taken on the role of pooling and managing funds for its own scheme, which includes pensions and civil servants’ health insurance, as well as the CBHI. This centralized fund arrangement has enhanced risk pooling. Despite having a smaller pool, the RSSB possesses a greater revenue base due to its members making higher contributions than CBHI members. Additionally, there is cross-subsidization from the RSSB to the CBHI and other insurance schemes, which, along with the consolidation of funds, has resulted in economies of scale [9,11].

Conversely, some countries have fragmented pools, which increase administrative costs and inefficiency. Both Tanzania and Kenya have several schemes and fragmented risk pools. For example, in Tanzania, the improved Community Health Fund (CHF), Tiba Kwa Kadi (IKA), and other micro-insurance schemes are voluntary, leading to low penetration and therefore limiting financial and risk-pooling [12]. In addition, the insurance schemes in Tanzania and Kenya cover different target groups rather than combine them. Therefore, they do not achieve efficiencies in scale and cross-subsidization [12,22]. Fragmented risk pools undermine risk-sharing, hinder efforts to widen insurance pools, and promote cross-subsidization. Most insurance pools remain small and, therefore, unsustainable in the long term.

While countries aim to enhance the redistribution of funds, options for pooling reform are context-specific and country-dependent. Countries should understand the multiple causes of fragmentation in their financing system and use this knowledge to define reform goals to gain efficiency [27]. For example, while in principle, the Social Health Insurance in Vietnam involves a single pool and all insurance schemes were merged, in practice, some challenges remain. There are 63 provincial pools covering populations ranging in size from 300,000 to 4.8 million people. The multitude of membership categories intensifies fragmentation since each category contributes differently to the overall risk pool. Risk pooling remains limited across insurance groups and provinces [28,29].

- Purchasing

This section focuses on 1) from which health providers do NHIs buy health services, and how do they choose them, and 2) how do they pay for their services. It highlights some of the strategies adopted and their challenges.

*From Whom and How to Purchase Services*: Countries assessed for this review purchase services from both public and private providers, with variations in each country. For example, Rwanda and Vietnam rely mainly on public providers, while Ghana, Indonesia, and Kenya purchase from both public and private providers with different arrangements.

Further, the countries reviewed showcase differences in their purchasing arrangements that can affect UHC and weaken their NHI system. For example, in Rwanda, under both the CBHI and RSSB schemes, all public facilities are automatically contracted to offer services included in the benefits package. These public facilities receive contracts of indefinite duration regardless of performance, which may impact service quality and efficiency. In addition, the RSSB aims to increase service accessibility by selectively contracting private health posts for the CBHI scheme and private facilities for specialized health services. While the RSSB uses selective contracting for private providers, all public providers are automatically included in both the CBHI and RSSB schemes. This policy of automatic contracting for public health facilities may reduce the motivation of managers to meet purchaser standards for service quality and efficiency [11].

In Vietnam, different purchasers can contract distinct types of providers. For example, both the Vietnam Social Security and the Ministry of Health can purchase services from central and provincial hospitals. However, services are paid using different mechanisms, leading to conflicting incentives [20]. The existence of multiple purchasers using different payment mechanisms can also undermine providers’ incentives. A study examining multiple funding flows highlights the potential impact of various payment streams on healthcare provider behavior. Evidence from six countries, including Kenya, Nigeria, and Vietnam, revealed that receiving multiple funding flows can prompt undesirable provider behaviors, potentially undermining efficiency, equity, and quality in service provision [30].

In Indonesia, the Social Security Agency of Health (SSAH or BPJS-K) can directly contract primary care providers and hospitals. The SSAH and its structures enable an adaptive system at scale to address the demands in a flexible manner (15). However, despite the set-up of a single purchaser of health services, Indonesia continues to have a mixed record in strategic purchasing due to decentralization. The decentralized system has strained the capacity of some local governments to plan and budget the provision of health services appropriately [31].

*How to Pay for Services–Provider Payment*: Most countries reviewed use fee-for-service as the dominant provider payment mechanism. Such a model entails fixed fees set in advance in exchange for each service or group of services delivered. The downside of fee-for-service is that it can incentivize an oversupply of services and quickly escalate costs. For example, in Rwanda, both the CBHI and RSSB schemes purchase health services using fee-for-service payments, setting the rates for providers at each level of public health facilities. Despite the introduction of new revenue sources, these payment methods have resulted in deficits [11].

Ghana, Kenya, and Vietnam have attempted to implement a capitation mechanism to contain costs. However, this payment system has had several challenges. In Ghana, contextual factors, which included the political climate at the time of the policy design and implementation, insufficient stakeholder involvement in the development of the policy, and inadequate understanding of the capitation mechanism by non-technical participants, led to a growing opposition to the policy and its subsequent suspension [32–34]. In Kenya, the provider payment mechanism introduced by the NHIF in 2015 failed to promote equity, efficiency, and quality healthcare services because the payment rates were seen as insufficient. Providers from both public and private facilities reported that the capitation rates for outpatient services were insufficient. They did not reflect the costs of services or the frequencies of NHIF beneficiaries’ visits to health facilities. As a result of the perceived inadequacy of the methods, beneficiaries were denied services, charged co-payment, and experienced long waiting times, among other negative effects [35].

Vietnam introduced a version of the capitation payment for district hospitals in 2009 under the Law on Health Insurance. However, the capitation payment did not yield the expected benefits for Vietnam’s health system. Under the model, the mechanism resulted in significant capitated rates among provinces and population groups, disconnected from health needs, and leading to equity concerns [28,36].

Purchasing is an important lever in UHC. It helps balance the costs and revenues of the system while creating incentives to deliver services more efficiently, which can in turn lead to expanded coverage. Strategic purchasing uses evidence and information about population health needs and health provider performance to make resource allocation decisions and improve access at an acceptable cost and level of quality. Countries reviewed could pursue a more strategic purchasing approach to enhance efficiency and equity [37]. For example, Ghana has advanced toward strategic purchasing, utilizing data on population needs and provider performance. As such, improvements have been implemented to align the benefits package with the burden of disease, engage in contracts with both public and private healthcare providers, introduce output-based payments to providers, and supervise the performance at both the provider and system levels [34].

### 3.3 Coverage

This section discusses the three dimensions of coverage defined by WHO. It identifies options and challenges associated with expanding population coverage and the benefits package. It also reviews the financial protection mechanisms implemented in the countries reviewed. Table 4 gives an overview of the coverage dimensions for the countries reviewed.

**Table 4:** Three dimensions of coverage in the countries reviewed.

*Country*	*Population*	*Population enrolled (% of total population)*	*Benefits package*	*UHC service coverage index (2019) †*	*Out-of-pocket expenditure as a % of THE (2020)* *
Ghana	Entire population targeted	~40%	Comprehensive	45	30.77
Kenya	Formal sector	~20%	Varies	56	24.06
Nigeria	Entire population targeted	>30%	Varies	44	74.68
Rwanda	Entire population targeted	>90%	Varies	54	10.34
Senegal	Entire population targeted	~46%	Varies	49	41.57
Tanzania	Formal sector	<20%	Varies	46	23.10
Zambia	Entire population targeted	~35%	Comprehensive	55	8.8
Cambodia	Formal sector	~30%	Comprehensive	61	60.60
Indonesia	Entire population targeted	>62%	Comprehensive	59	31.79
Philippines	Entire population targeted	>80%	Comprehensive	55	45.03
Vietnam	Entire population targeted	>60%	Comprehensive	70	39.60

*Abbreviations*: *UHC: Universal Health Coverage; THE: Total Health Expenditures; OOP: Out-of-Pocket*

† *This indicator is an index reported on a unit-less scale of 0 to 100, which is computed as the geometric mean of 14 tracer indicators of health service coverage*. *The tracer indicators are as follows, organized by four components of service coverage: 1. Reproductive, maternal, newborn, and child health 2. Infectious diseases 3. Non-communicable diseases 4. Service Capacity and Access*

* This indicator is of critical importance in assessing the extent of financial protection within a country. *If out-of-pocket (OOP) expenditure is a high percentage of total health expenditure*, *this will generally suggest limited financial protection*. *WHO recommends that total out-of-pocket payments should not exceed 15–20% of national health expenditures to reduce the incidence of catastrophic health payments* [12].

- Population covered: expanding enrollment

Several interlinked factors help explain the degree of coverage of a specific population, one of them being whether enrollment in a health insurance scheme is mandatory or voluntary. Evidence shows that when enrollment and premium contributions are voluntary, population coverage is generally low [12,22]. For example, in Kenya where health insurance in the informal sector is voluntary, coverage is low [38]. In 2018, the National Health Insurance Fund (NHIF) was the main insurer for the formal sector covering 16 per cent of the population, whereas the 32 private health insurers collectively covered only about 1 per cent of the Kenyan population [22,32]. Likewise, in Nigeria, the voluntary nature of the NHI scheme has led to poor enrollment [39]. In Cambodia, data from 2020 revealed that under the country’s NSSF voluntary scheme, 53 per cent of the population lacked any social health protection coverage, and approximately 16 per cent of those legally eligible for a scheme had not yet enrolled to receive benefits [40].

However, mandatory enrollment is not a sufficient condition for success. In Ghana, the legislation makes it mandatory for every Ghanaian to register in the NHIS. While about 40 per cent of the population is enrolled, this figure has remained stagnant [10,18]. The Ghana experience shows how enforcing a mandatory enrollment can be difficult and that several other conducive factors are needed to ensure important levels of enrollment. Rwanda has distinguished itself as the sole country in Sub-Saharan Africa to attain enrollment rates exceeding 80 per cent of the population [11]. Similar to Ghana, membership in Rwanda is mandatory. However, unlike Ghana, Rwanda has enforced enrollment through fines that amount to between 50,000 and 100,000 Rwandan Franc (USD 45 and 90) [9]. In Ghana, the legislation does not foresee a penalty for non-enrollees [41], which may be one of the reasons why population coverage has not progressed further.

To expand coverage, the examples of Ghana and Rwanda show that other factors beyond compulsory enrollment are necessary. Challenges include the high costs and unaffordability of premium contributions, the perception among parts of the population that health insurance is meant only for the sick or specific groups such as children and pregnant women, a lack of trust in the scheme’s operators, the poor quality of services provided, and a lack of information and education regarding the principles of risk-sharing [35,41–43].

Further, good governance and accountability have been key in expanding coverage in Rwanda. During the first decade of Rwanda’s CBHI, the percentage of the population covered rose from 1 to about 90 per cent [44,45]. The Rwandan government made local officials the mainstay of the expansion by making them accountable through performance evaluations. District coverage levels were a key indicator in a performance-based financing scheme, also called the *Imihigo* system. In addition, the law specified that an individual was eligible for health coverage only when every member of the household was enrolled [9,45].

Several interlinked factors are at play when ensuring and expanding enrollment. Firstly, mandatory enrollment does not automatically translate into high population coverage. At the same time, the contributory nature of an insurance scheme can have negative implications regarding equity, access to healthcare, and financial protection, especially for LLMICs. One drawback is that it often only covers individuals who can afford to pay, excluding those who need health coverage the most (e.g., individuals with low incomes, informal workers, and unemployed people) [24]. Secondly, the decision to enroll in and use health insurance is strongly influenced by perceptions, education, and cultural factors. To engage participants and ensure enrollment, it is essential to have a robust, coordinated, and continuous effort to inform and educate people on the role and importance of health insurance. Increased awareness and understanding of the potential benefits of insurance coverage by the population are paramount. Finally, whether the scheme is mandatory or voluntary, beneficiaries should be able to access quality healthcare and be satisfied with the services received.

While population coverage may indicate progress toward UHC, WHO has recently used two dimensions to assess UHC: service coverage and financial protection [1]. Indeed, high population coverage may not automatically translate into available and affordable health services. For example, a study in Indonesia revealed that while insurance coverage was most extensive in poorer regions, physical access to services was low (merely 27 per cent of villages in poorer regions had convenient access to a hospital, compared to 93 per cent in richer areas [46].

- Benefits package: which services to provide while ensuring provision

One of the dimensions of UHC is the comprehensiveness of the services offered to the population and the fact that they are covered by insurance. Countries reviewed have opted for different strategies: a unique and comprehensive benefits package, a comprehensive package but with different services across insured groups, or a limited package that varies across covered groups. In terms of cost-sharing arrangements, no co-payment at the point of care is required for any users in some countries, such as Ghana. In others, only the very poor are exempted from co-payment, such as in Vietnam. Table 5 showcases the type of benefits package, the services included, and cost-sharing arrangements.

**Table 5:** Overview of selected country’s benefits package.

*Country*	*Benefits package*	*Services included under the scheme*	*Cost-sharing arrangements*	*References*	
Ghana	Comprehensive and universal	Out-patient and in-patient services, maternity care, eye care, and oral healthcare services.	No cost-sharing requirements.	[10,18,47,48]	
Kenya	Varies with the insurance scheme	*Under the national scheme (previously known as the National Health Insurance Fund (NHIF)*: outpatient services, specialized services including renal dialysis and kidney transplant; radiology and oncology packages; chronic illness; maternity package; rehabilitation package; specialized laboratory tests; surgical package (major, minor and specialized); foreign treatment; and emergency evacuation services.	Cost-sharing in public health facilities.	[22,35,38]	
Nigeria	Varies depending on the groups covered	*Under the Formal Sector Social Health Insurance Programme (FSSHIP)*: standard benefits package that specifies primary, secondary, and tertiary services, with some exclusions for high technology investigations and occupational diseases.	Packages do not specify any cost-sharing arrangements or limits. Providers charge user fees for services.	[49,50]	
Rwanda	Varies according to the scheme (RSBB or CBHI)	CBHI members have access to a comprehensive range of preventive, rehabilitative, and curative services and drugs offered by public health facilities and some private health facilities. Members of the RSSB scheme have access to all services offered by public health facilities, as well as specialized services provided by private health providers.		[11,51]	
	Indonesia	Comprehensive and universal	Outpatient and inpatient services, including maternal and child health services, prescribed laboratory tests, dental healthcare, ambulance services for referrals, and advanced health services (e.g. oncology package and hemodialysis).	Free access to primary healthcare facilities when sick. Referrals are needed to access secondary and tertiary facilities.	[52,53]
	Philippines	Comprehensive and universal	Outpatient with Primary Care Benefits (PCB): PCB I covers doctors and diagnostics, and PCB II covers outpatient pharmaceuticals; Inpatient care regardless of membership type.	The current NHIP benefit scheme covers all medical costs for inpatient care but only up to specified benefit ceilings.	[54,55]
	Vietnam	Comprehensive and universal	Include curative and preventive healthcare services: medical examination, treatment, functional rehabilitation, pregnancy checkups and delivery, screening, and early diagnosis of some diseases.	Poor and ethnic groups are exempted from payment.	[29,56,57]

The nature and comprehensiveness of the benefits package are expected to influence access and utilization of health services for the population that is covered by national health insurance. One assumes that the more inclusive of services the package is, the better is access and therefore, utilization. The review shows that for the countries assessed, despite a comprehensive and inclusive package of services in some countries, several systemic challenges may hinder access and especially the utilization of health services. Some of these challenges include:

- lack of human resources, infrastructure, and uneven geographic distribution of services;
- poor communication of the benefits package, limiting access to and utilization of services;
- variation in entitlements, contributing to inequitable access and utilization.

Table 6 highlights some examples of the key challenges observed in the countries reviewed.

**Table 6:** Examples of challenges related to the benefits package.

***Human resources for health*, *infrastructure*, *and geographical distribution***
In Ghana, the geographic distribution of healthcare facilities and personnel was a challenge to access healthcare and, therefore, to the successful implementation of the scheme [34,58].
In Kenya, following the health reforms undertaken in 2015, the range of benefits that members received was limited due to the unavailability of certain services from the healthcare providers contracted by the NHIF [22,35,59]. In addition, the selection and contracting of providers was characterized by pro-urban and pro-private distribution, leading to inequities in healthcare access. In addition, the new benefit entitlements were not accompanied by infrastructure improvements, resulting in service delivery infrastructure gaps in public healthcare facilities [35].
In Indonesia, while the prosperous region had the lowest health needs and moderate insurance coverage, it enjoyed better access to services and had a higher healthcare utilization. In contrast, the country’s poorer provinces (Eastern Indonesia) had the highest health needs and insurance coverage but also the lowest access to healthcare services and lower utilization [46].
***Communication of the benefits package***
In Nigeria, providers’ lack of knowledge and awareness surrounding the scheme affected enrollment and coverage [39].
In Kenya, following the 2015 health reforms, poor communication to both providers and beneficiaries led to a poor understanding of entitlements’ specifications and new benefits included, particularly among disadvantaged groups [35,59].
***Entitlement differences***
In Rwanda, the CBHI and RSSB schemes offer different benefits packages. The RSSB scheme has a broader benefits package and a wider range of contracted providers than CBHI [11].
In Tanzania, members of the NHIF, who benefit from better access, were more likely than CHF members to seek care and were also less likely to delay seeking care [60].

While improvements in service coverage have been reported in the countries reviewed by the Global Monitoring Report (2023) [1], health authorities must devise a benefits package that fits the country’s needs and constraints. Firstly, building adequate infrastructure and provider capacity is paramount to address supply-side constraints and deliver quality healthcare services. This is particularly relevant in poorer regions within a country, where health needs tend to be higher [46]. Finally, a balanced distribution of private/public–urban/rural healthcare providers will lead to enhanced access by the population.

Secondly, explicitly defining the benefit package leads to a better understanding of service entitlements by members and providers. Good examples include lists specifying which services are included and/or excluded. Communicating changes in the benefits package in a timely manner ensures that beneficiaries get the expected entitlements and that providers offer all covered services.

Finally, the existence of a harmonized benefit package among different existing schemes can mitigate inequities between groups. Insured members should enjoy equal access to healthcare services irrespective of their ability to pay or affiliation to an insurance scheme. A differentiated benefit package is likely to result in different degrees of access and outcomes.

- Financial protection: subsidizing the most vulnerable while ensuring equity

The third objective of UHC is to protect the population from financial hardship. The ability to offer adequate financial risk protection depends on the government’s commitment to develop a national financing strategy and health schemes that protect the most vulnerable. This section provides an overview of relevant mechanisms implemented to protect households and individuals from financial hardship in the countries reviewed.

Under their universal scheme, Ghana, Zambia, and Indonesia provide premium exemptions to specific groups of the population that do not need to contribute [18,52,53,61,62].

In Kenya, the Health Insurance Subsidy for the Poor (HISP) program under the NHIF is a comprehensive, fully subsidized health insurance program linked to the government’s cash transfer program. HISP beneficiaries receive comprehensive services from contracted public and private providers [22,35]. In addition, Kenya has the Linda Mama Free maternity scheme, which is automatic and mandatory for all pregnant women who are Kenyan citizens [35]. Senegal has also implemented a few different coverage programs targeting the most vulnerable populations, including the poorest households, people with disabilities, school-age children, people over the age of 60 years old, and caesarean sections [63]. In Nigeria, the Ministry of Health attempts to regulate user fees and drug charges for health services to remain affordable to vulnerable populations. For example, it provides free maternal and child healthcare services for pregnant women and children under five years of age [49].

In the Philippines, the Sponsored Program integrated into the National Health Insurance scheme offers full subsidies, financed by the Sin Tax, to aid the most vulnerable, the near-poor [54], the indigents, and older adults [26]. Similarly, Vietnam set up the Health Care Fund for the Poor (HCFP) and provided subsidies for premium payments to assist the poor and near-poor. In 2014, the government implemented the Revised Health Insurance Law, which modified copayment rates. As a result, groups such as the poor, ethnic minorities, policy beneficiaries like war veterans, and residents of socio-economically difficult areas and islands were exempted from copayment and granted access to free medical services. Additionally, the copayment rate for the near-poor was decreased from 20 to 5 per cent [29].

While the national health insurance scheme in Cambodia targets the formal sector, the country also provides a voluntary health scheme for the near-poor and informal sector [64]. Under the Health Equity Fund (HEF), all HEF beneficiaries are provided all healthcare services by individual health facilities at no out-of-pocket cost at any level of the health system [65].

Similarly, in Tanzania, the Community Health Fund (CHF) is a voluntary scheme targeting the informal sector workers. A large part of the Tanzanian population falls within this category, and this community fund relies on household premiums and national government contributions. Households considered too impoverished are not required to contribute [66]. However, only a quarter of the population is enrolled in the CHF, attributed to a lack of understanding among potential beneficiaries, a limited benefits package, and a poor quality of care in public health facilities [60,66].

To ensure financial equity, Rwanda adopted the principle of stratification of premiums according to beneficiaries’ wealth, leveraging information from the Ubudehe program. The program assesses the socioeconomic status of citizens to implement a progressive tiered premium collection system, offering full subsidies for members of the two lowest wealth sub-categories. Ubudehe classification considers income, household assets, and work capacity based on the collective assessment by village members. The Ministry of Health has utilized this initiative to modulate the premium of the Mutuelles [9,45].

Table 7 provides a more exhaustive list of groups exempted from contributions or that are fully subsidized.

**Table 7:** Financial protection mechanisms.

*Country*	*Scheme or Program*	*Type*	*Targeted population*	*Mechanism*	*References*
Ghana	NHIS	Universal	Formal sector employees and the self-employed who contribute to the Social Security and National Insurance Trust; children (under 18 years of age); pregnant women and post-natal healthcare services; indigents; categories of differently-abled persons; persons with mental disorders; pensioners of the Social Security and National Insurance Trust; persons above seventy years of age	Premium exemptions	[47]
Kenya	Health Insurance Subsidy for the Poor (HISP) under the NHIF	Targeted	Households with orphans and vulnerable children; poor elderly; persons with disabilities and destitute families	Fully subsidized by the government’s cash transfer program	[22,35]
Nigeria	Various	Targeted	The most vulnerable	Varies	[49]
Rwanda	CBHI	Universal	Members of the two poorest sub-categories	Fully subsidized	[9,45]
Senegal	Various	Targeted	The most vulnerable	Varies	[63]
Zambia	NHI	Universal	Individuals over 65 years of age and under 18 years	Premium exemptions	[32,61]
Cambodia	Health Equity Fund (HEF)	Targeted	Three beneficiary categories: (1) the poor as identified by the ID Poor system;	Full government subsidies from the poor to informal workers above the poverty line	[64,65]
			(2) informal workers, defined as people working less than eight hours per day or seasonal workers; and		
			(3) special category beneficiaries, which include commune council members, deputy village chiefs, village chiefs, professional sports practitioners, and association members		
Indonesia	JKN	Universal	The poor and near poor (classified as Subsidized Contribution Recipients or non-contributory beneficiaries)	Premium exemptions subsidized by both central and local governments	[52,53]
The Philippines	Sponsored Program (under the National Health Insurance Program)	Targeted	The most vulnerable, the near poor, the indigent, and older adults	Fully subsidized and financed by the Sin Tax	[26,54,55]
Vietnam	Health Care Fund for the Poor (HCFP)	Targeted	The poor and near-poor; other groups living in socio-economically difficult areas and islands	Subsidized by State budget	[29,57]

Financial protection is achieved when there are no financial barriers to accessing needed health services, and OOP health expenditures are not a source of financial hardship. However, we observe that the OOP health expenditures remain above the 15–20 percent threshold recommended by the WHO [12] (Table 4). Indicators for Cambodia and Nigeria are particularly high despite strategies to protect the most vulnerable [17]. In addition, for both countries, the estimated proportion of the population with OOP health spending exceeding 10 per cent of the household budget is between 10 and 20 per cent [1]. Overall, for all countries, despite mechanisms to protect the most vulnerable, further strategies are needed to limit the level of catastrophic health expenditures, especially since the latest evidence from the Global Monitoring Report (2023) indicates an increasing trend in both Sub-Saharan Africa and South Asia [1].

### 3.4 Strengths and limitations

A few limitations to this review should be acknowledged. First, not all countries reviewed had the available information that the study aimed to assess in a systematic manner, and the limited evidence found for several countries may limit comparison. Second, this review aimed to review countries with a running national health insurance scheme or program, and countries were selected intentionally. Therefore, there is subjectivity in countries’ selection. Third, the countries reviewed present varied income levels and health expenditures and are at different phases of their national health insurance scheme implementation. Therefore, any comparison of their performance should be approached with caution. Finally, it should be noted that using an appraisal tool for the selection of studies might have informed different findings.

Despite these limitations, we believe this review provides valuable information for LLMICs considering strategies and best practices to progress toward universal health coverage under a national health insurance program. The review provides lessons drawing from countries’ experiences by offering evidence-informed approaches to progress on the UHC goals.

## 4. Conclusion and recommendations

This review assesses how LLMICs that have implemented an NHIS raise, manage, and use funds; expand coverage; provide and increase services; and protect the most vulnerable. Findings show that countries adopt different strategies to progress toward UHC. First, in terms of health financing functions, countries generate revenue through various public and private means, including general and earmarked taxes, payroll taxes, premiums, and out-of-pocket payments. Some countries have consolidated their revenue streams into a single pool to enhance equity and efficiency. In contrast, others maintain separate pools, leading to higher administrative costs. Healthcare services are procured from public and private providers, differing by country. Fee-for-service is the prevalent payment method, but a few countries have attempted capitation systems to control expenses. Second, population coverage depends on whether enrollment in an NHIS is mandatory or voluntary, and on its enforcement. Service provision can be included in a unique and comprehensive benefits package; a comprehensive package offering different services across insured groups; or a limited package with variations for the covered groups. Finally, mechanisms to avoid financial hardship can involve premium exemptions or government subsidies.

Progressing toward UHC requires responding to critical questions. Countries should ensure financial sustainability, guarantee cost-containment, and expand and maintain population enrollment while safeguarding financial and health equity. We provide some recommendations based on the strategies assessed. Countries should consider sustainable ways of collecting revenue, which may include increasing the allocation of tax revenues to the insurance scheme. Then, they should consider merging risk pools incrementally by reducing the number of insurance categories. Finally, countries should adopt strategic purchasing to enhance performance, providers’ incentives, and establish provider payment mechanisms that ensure cost containment. In terms of the coverage dimensions, countries should emphasize the importance of communicating the scheme’s benefits to retain and expand enrollment, especially if it is a voluntary scheme. Regarding the benefits package, countries should ensure adequate provider capacity, infrastructure investments, a balanced geographical distribution of providers, and quality services. They should also have communication strategies on service entitlements to ensure health utilization. The benefits package should be harmonized across schemes to safeguard equity. Finally, catastrophic health expenditures need to be reduced to protect the most vulnerable.

Although policy options are context-specific and country-dependent, lessons deriving from this review can be used to inform approaches for improving universal health coverage.

## Supporting information

S1 Table PRISMA ScR checklist. (DOCX)

S1 Fig PRISMA flow diagram. (DOCX)

S1 Data Links to articles. (DOCX)
