# Supplementary material for: Moving toward universal health coverage with a national health insurance program: A scoping review and narrative synthesis of experiences in eleven low- and lower-middle income countries
Source: PLOS Glob Public Health. 2025 Jan 9;5(1):e0003651. doi: 10.1371/journal.pgph.0003651 (PMC11717203; doi:10.1371/journal.pgph.0003651)
Supplement: S1 Fig — (DOCX) [file pgph.0003651.s002.docx]

**Identification of studies via other methods**

**Identification of studies via databases and registers**

Records identified from:

Websites (n = 9)

Organisations (n = 8)

Citation searching (n = 5)

Records removed *before screening*:

Duplicate records removed (n = 263)

Records marked as ineligible by automation tools (n = 0)

Records removed for other reasons (n = 0)

Records identified from:

Databases total (n = 569)

Registers (n = 0)

**Identification**

Records excluded

(n = 123)

Records screened

(n = 306)

Reports not retrieved

(n = 5)

Reports sought for retrieval

(n = 22)

Reports not retrieved

(n = 84)

Reports sought for retrieval

(n = 183)

**Screening**

Reports excluded:

(n = 7)

Reports assessed for eligibility

(n = 17)

Reports excluded:

(n = 31)

Reports assessed for eligibility

(n = 99)

Studies included in review (n = 68)

Reports of included studies (n = 10)

**Included**
