# Supplementary material for: Moving toward universal health coverage with a national health insurance program: A scoping review and narrative synthesis of experiences in eleven low- and lower-middle income countries
Source: PLOS Glob Public Health. 2025 Jan 9;5(1):e0003651. doi: 10.1371/journal.pgph.0003651 (PMC11717203; doi:10.1371/journal.pgph.0003651)
Supplement: S1 Data — (DOCX) [file pgph.0003651.s003.docx]

Abiiro, G. A., Alatinga, K. A., & Yamey, G. (2021). Why did Ghana’s national health insurance capitation payment model fall off the policy agenda? A regional level policy analysis. *Health Policy and Planning*, *36*(6), 869–880. <https://doi.org/10.1093/heapol/czab016>

Abuya, T., Maina, T., & Chuma, J. (2015). Historical account of the national health insurance formulation in Kenya: Experiences from the past decade. *BMC Health Services Research*, *15*(1), 56. <https://doi.org/10.1186/s12913-015-0692-8>

Agustina, R., Dartanto, T., Sitompul, R., Susiloretni, K. A., Suparmi, Achadi, E. L., Taher, A., Wirawan, F., Sungkar, S., Sudarmono, P., Shankar, A. H., Thabrany, H., Agustina, R., Dartanto, T., Sitompul, R., Susiloretni, K. A., Suparmi, Achadi, E. L., Taher, A., … Khusun, H. (2019). Universal health coverage in Indonesia: Concept, progress, and challenges. *The Lancet*, *393*(10166), 75–102. <https://doi.org/10.1016/S0140-6736(18)31647-7>

Alawode, G. O., & Adewole, D. A. (2021). Assessment of the design and implementation challenges of the National Health Insurance Scheme in Nigeria: A qualitative study among sub-national level actors, healthcare and insurance providers. *BMC Public Health*, *21*(1), 124. <https://doi.org/10.1186/s12889-020-10133-5>

Amporfu, E., Agyei-Baffour, P., Edusei, A., Novignon, J., & Arthur, E. (2022). Strategic Health Purchasing Progress Mapping: A Spotlight on Ghana’s National Health Insurance Scheme. *Health Systems & Reform*, *8*(2), e2058337. <https://doi.org/10.1080/23288604.2022.2058337>

Asante, A., Cheng, Q., Susilo, D., Satrya, A., Haemmerli, M., Fattah, R. A., Kosen, S., Novitasari, D., Puteri, G. C., Adawiyah, E., Hayen, A., Mills, A., Tangcharoensathien, V., Jan, S., Thabrany, H., & Wiseman, V. (2023). The benefits and burden of health financing in Indonesia: Analyses of nationally representative cross-sectional data. *The Lancet Global Health*, *11*(5), e770–e780. <https://doi.org/10.1016/S2214-109X(23)00064-5>

Atim, C., Bhushan, I., Blecher, M., Gandham, R., Rajan, V., Davén, J., & Adeyi, O. (2021). Health financing reforms for Universal Health Coverage in five emerging economies. *Journal of Global Health*, *11*, 16005. <https://doi.org/10.7189/jogh.11.16005>

Barasa, E., Rogo, K., Mwaura, N., & Chuma, J. (2018). Kenya National Hospital Insurance Fund Reforms: Implications and Lessons for Universal Health Coverage. *Health Systems & Reform*, *4*(4), 346–361. <https://doi.org/10.1080/23288604.2018.1513267>

Barroy, H., Jarawan, E., & Bales, S. (2014). *Vietnam: Learning from Smart Reforms on the Road to Universal Health Coverage*. <http://hdl.handle.net/10986/20739>

Chemouni, B. (2018). The political path to universal health coverage: Power, ideas and community-based health insurance in Rwanda. *World Development*, *106*, 87–98. <https://doi.org/10.1016/j.worlddev.2018.01.023>

Chomi, E., Mujinja, P. G., Enemark, U., Hansen, K., & Kiwara, A. (2014). Health care seeking behaviour and utilisation in a multiple health insurance system: Does insurance affiliation matter? *International Journal for Equity in Health*, *13*(1), 25. <https://doi.org/10.1186/1475-9276-13-25>

Christmals, C. D., & Aidam, K. (2020). Implementation of the National Health Insurance Scheme (NHIS) in Ghana: Lessons for South Africa and Low- and Middle-Income Countries. *Risk Management and Healthcare Policy*, *13*, 1879–1904. <https://doi.org/10.2147/RMHP.S245615>

Dkhimi, F., Honda, A., Hanson, K., Mbau, R., Onwujekwe, O., Phuong, H. T., Mathauer, I., Akhnif, E. H., Jaouadi, I., Kiendrébéogo, J. A., Ezumah, N., Kabia, E., & Barasa, E. (2023). Examining multiple funding flows to public healthcare providers in low- and middle-income countries—Results from case studies in Burkina Faso, Kenya, Morocco, Nigeria, Tunisia and Vietnam. *Health Policy and Planning*, *38*(10), 1139–1153. <https://doi.org/10.1093/heapol/czad072>

Ezenwaka, U., Gatome-Munyua, A., Nwankwor, C., Olalere, N., Orji, N., Ewelike, U., Uzochukwu, B., & Onwujekwe, O. (2022). Strategic Health Purchasing in Nigeria: Investigating Governance and Institutional Capacities within Federal Tax-Funded Health Schemes and the Formal Sector Social Health Insurance Programme. *Health Systems & Reform*, *8*(2), e2074630. <https://doi.org/10.1080/23288604.2022.2074630>

Fenny, A. P., Yates, R., & Thompson, R. (2021). Strategies for financing social health insurance schemes for providing universal health care: A comparative analysis of five countries. *Global Health Action*, *14*(1), 1868054. <https://doi.org/10.1080/16549716.2020.1868054>

Fusheini, A., Marnoch, G., & Gray, A. M. (2017). Implementation Challenges of the National Health Insurance Scheme in Selected Districts in Ghana: Evidence from the Field. *International Journal of Public Administration*, *40*(5), 416–426. <https://doi.org/10.1080/01900692.2015.1127963>

Ha, B. T. T., Frizen, S., Thi, L. M., Duong, D. T. T., & Duc, D. M. (2014). Policy processes underpinning universal health insurance in Vietnam. *Global Health Action*, *7*(1), 24928. <https://doi.org/10.3402/gha.v7.24928>

Ibe, O., Honda, A., Etiaba, E., Ezumah, N., Hanson, K., & Onwujekwe, O. (2017). Do beneficiaries’ views matter in healthcare purchasing decisions? Experiences from the Nigerian tax-funded health system and the formal sector social health insurance program of the National Health Insurance Scheme. *International Journal for Equity in Health*, *16*(1), 216. <https://doi.org/10.1186/s12939-017-0711-y>

Kabia, E., Kazungu, J., & Barasa, E. (2022). The Effects of Health Purchasing Reforms on Equity, Access, Quality of Care, and Financial Protection in Kenya: A Narrative Review. *Health Systems & Reform*, *8*(2), 2114173. <https://doi.org/10.1080/23288604.2022.2114173>

Kaiser, A. H., Rotigliano, N., Flessa, S., Ekman, B., & Sundewall, J. (2023). Extending universal health coverage to informal workers: A systematic review of health financing schemes in low- and middle-income countries in Southeast Asia. *PloS One*, *18*(7), e0288269. <https://doi.org/10.1371/journal.pone.0288269>

Kalisa, I., Musange, S., Collins, D., Saya, U., & Kunda, T. (2015). *The Development of Community-Based Health Insurance in Rwanda—Experiences and Lessons* (p. 70). University of Rwanda College of Medicine and Health Sciences - School of Public Health, Kigali, Rwanda and Management Sciences for Health, Medford, MA, USA. <https://msh.org/wp-content/uploads/2016/04/the_development_of_cbhi_in_rwanda_experiences_and_lessons.pdf>

Kolesar, R. (2019). *Comparing Social Health Protection Schemes in Cambodia*. <http://www.healthpolicyplus.com/pubs.cfm?get=11281>

Kolesar, R. J., Pheakdey, S., Jacobs, B., Chan, N., Yok, S., & Audibert, M. (2020). Expanding social health protection in Cambodia: An assessment of the current coverage potential and gaps, and social equity considerations. *International Social Security Review*, *73*(1), 35–63. <https://doi.org/10.1111/issr.12227>

Kozhimannil, K. B., Valera, M. R., Adams, A. S., & Ross-Degnan, D. (2009). The population-level impacts of a national health insurance program and franchise midwife clinics on achievement of prenatal and delivery care standards in the Philippines. *Health Policy*, *92*(1), 55–64. <https://doi.org/10.1016/j.healthpol.2009.02.009>

Lu, C., Chin, B., Lewandowski, J. L., Basinga, P., Hirschhorn, L. R., Hill, K., Murray, M., & Binagwaho, A. (2012). Towards Universal Health Coverage: An Evaluation of Rwanda Mutuelles in Its First Eight Years. *PLoS ONE*, *7*(6), e39282. <https://doi.org/10.1371/journal.pone.0039282>

Ly, M. S., Bassoum, O., & Faye, A. (2022). Universal health insurance in Africa: A narrative review of the literature on institutional models. *BMJ Global Health*, *7*(4), e008219. <https://doi.org/10.1136/bmjgh-2021-008219>

Makaka, A., Breen, S., & Binagwaho, A. (2012). Universal health coverage in Rwanda: A report of innovations to increase enrolment in community-based health insurance. *The Lancet*, *380*, S7. <https://doi.org/10.1016/S0140-6736(13)60293-7>

Mao, W., Tang, Y., Tran, T., Pender, M., Khanh, P. N., & Tang, S. (2020). Advancing universal health coverage in China and Vietnam: Lessons for other countries. *BMC Public Health*, *20*(1), 1791. <https://doi.org/10.1186/s12889-020-09925-6>

Mathauer, I., Vinyals Torres, L., Kutzin, J., Jakab, M., & Hanson, K. (2020). Pooling financial resources for universal health coverage: Options for reform. *Bulletin of the World Health Organization*, *98*(2), 132–139. <https://doi.org/10.2471/BLT.19.234153>

Maulana, N., Soewondo, P., Adani, N., Limasalle, P., & Pattnaik, A. (2022). How Jaminan Kesehatan Nasional (JKN) coverage influences out-of-pocket (OOP) payments by vulnerable populations in Indonesia. *PLOS Global Public Health*, *2*(7), e0000203. <https://doi.org/10.1371/journal.pgph.0000203>

Mbau, R., Kabia, E., Honda, A., Hanson, K., & Barasa, E. (2020). Examining purchasing reforms towards universal health coverage by the National Hospital Insurance Fund in Kenya. *International Journal for Equity in Health*, *19*(1), 19. <https://doi.org/10.1186/s12939-019-1116-x>

Ministry of Health. (2015). *Ghana Health Financing Strategy* (p. 70). Ministry of Health. <https://www.moh.gov.gh/wp-content/uploads/2016/02/Health-Finance-Strategy-160203045304.pdf>

NHIS. (n.d.-a). *About Us*. Brief Introduction to the NHIS. Retrieved February 27, 2023, from <https://nhis.gov.gh/about>

NHIS. (n.d.-b). *Benefits Package*. Benefits Package. Retrieved February 24, 2023, from <https://www.nhis.gov.gh/benefits>

Novignon, J., Lanko, C., & Arthur, E. (2021). Political economy and the pursuit of universal health coverage in Ghana: A case study of the National Health Insurance Scheme. *Health Policy and Planning*, *36*(Supplement_1), i14–i21. <https://doi.org/10.1093/heapol/czab061>

Nyandekwe, M., Nzayirambaho, M., & Kakoma, J. B. (2020). Universal health insurance in Rwanda: Major challenges and solutions for financial sustainability case study of Rwanda community-based health insurance part I. *Pan African Medical Journal*, *37*. <https://doi.org/10.11604/pamj.2020.37.55.20376>

Obermann, K., Jowett, M., & Kwon, S. (2018). The role of national health insurance for achieving UHC in the Philippines: A mixed methods analysis. *Global Health Action*, *11*(1), 1483638. <https://doi.org/10.1080/16549716.2018.1483638>

Osei Afriyie, D., Hooley, B., Mhalu, G., Tediosi, F., & Mtenga, S. M. (2021). Governance factors that affect the implementation of health financing reforms in Tanzania: An exploratory study of stakeholders’ perspectives. *BMJ Global Health*, *6*(8), e005964. <https://doi.org/10.1136/bmjgh-2021-005964>

Osei Afriyie, D., Krasniq, B., Hooley, B., Tediosi, F., & Fink, G. (2022). Equity in health insurance schemes enrollment in low and middle-income countries: A systematic review and meta-analysis. *International Journal for Equity in Health*, *21*(1), 21. <https://doi.org/10.1186/s12939-021-01608-x>

Phuong, N. K., Oanh, T. T. M., Phuong, H. T., Tien, T. V., & Cashin, C. (2015). Assessment of systems for paying health care providers in Vietnam: Implications for equity, efficiency and expanding effective health coverage. *Global Public Health*, *10*(sup1), S80–S94. <https://doi.org/10.1080/17441692.2014.986154>

Pratiwi, A. B., Setiyaningsih, H., Kok, M. O., Hoekstra, T., Mukti, A. G., & Pisani, E. (2021). Is Indonesia achieving universal health coverage? Secondary analysis of national data on insurance coverage, health spending and service availability. *BMJ Open*, *11*(10), e050565. <https://doi.org/10.1136/bmjopen-2021-050565>

Roll, A., Katz, A., & Lane, J. (2021). A framework policy analysis of national health insurance policymaking in sub-Saharan Africa. *Health Policy and Planning*, *36*(8), 1246–1256. <https://doi.org/10.1093/heapol/czaa160>

Sarkodie, A. O. (2021). Effect of the National Health Insurance Scheme on Healthcare Utilization and Out-of-Pocket Payment: Evidence from GLSS 7. *Humanities and Social Sciences Communications*, *8*(1), Article 1. <https://doi.org/10.1057/s41599-021-00984-7>

Sinjela, K. M., Simangolwa, W. M. W., Hehman, L., Kamanga, M., Mwambazi, W. K., & Sundewall, J. (2022). Exploring for-profit healthcare providers’ perceptions of inclusion in the Zambia National Health Insurance Scheme: A qualitative content analysis. *PLOS ONE*, *17*(5), e0268940. <https://doi.org/10.1371/journal.pone.0268940>

Siongco, K. L. L., Nakamura, K., & Seino, K. (2020). Reduction in inequalities in health insurance coverage and healthcare utilization among older adults in the Philippines after mandatory national health insurance coverage: Trend analysis for 2003–2017. *Environmental Health and Preventive Medicine*, *25*(1), 17. <https://doi.org/10.1186/s12199-020-00854-9>

Somanathan, A., Tandon, A., Dao, H. L., Hurt, K. L., & Fuenzalida-Puelma, H. L. (2014). *Moving toward Universal Coverage of Social Health Insurance in Vietnam: Assessment and Options*. The World Bank. <https://doi.org/10.1596/978-1-4648-0261-4>

Suchman, L. (2018). Accrediting private providers with National Health Insurance to better serve low-income populations in Kenya and Ghana: A qualitative study. *International Journal for Equity in Health*, *17*(1), 179. <https://doi.org/10.1186/s12939-018-0893-y>

Taverne, B., Laborde-Balen, G., Diaw, K., Gueye, M., Have, N.-N., Etard, J.-F., & Sow, K. (2021). Does universal health coverage reduce out-of-pocket expenditures for medical consultations for people living with HIV in Senegal? An exploratory cross-sectional study. *BMJ Open*, *11*(7), e046579. <https://doi.org/10.1136/bmjopen-2020-046579>

Thuong, N. T. T., Huy, T. Q., Tai, D. A., & Kien, T. N. (2020). Impact of Health Insurance on Health Care Utilisation and Out-of-Pocket Health Expenditure in Vietnam. *BioMed Research International*, *2020*, 1–16. <https://doi.org/10.1155/2020/9065287>

Tobe, M., Stickley, A., Del Rosario, R. B., & Shibuya, K. (2013). Out-of-pocket medical expenses for inpatient care among beneficiaries of the National Health Insurance Program in the Philippines. *Health Policy and Planning*, *28*(5), 536–548. <https://doi.org/10.1093/heapol/czs092>

Umuhoza, S. M., Musange, S. F., Nyandwi, A., Gatome-Munyua, A., Mumararungu, A., Hitimana, R., Rulisa, A., & Uwaliraye, P. (2022). Strengths and Weaknesses of Strategic Health Purchasing for Universal Health Coverage in Rwanda. *Health Systems & Reform*, *8*(2), e2061891. <https://doi.org/10.1080/23288604.2022.2061891>

Wang, H., Otoo, N., & Dsane-Selby, L. (2017). *Ghana National Health Insurance Scheme: Improving Financial Sustainability Based on Expenditure Review*. World Bank. <https://doi.org/10.1596/978-1-4648-1117-3>

WHO. (2019, March 22). *Purchasing health services for universal health coverage: How to make it more strategic?* <https://www.who.int/publications-detail-redirect/WHO-UCH-HGF-PolicyBrief-19.6>

WHO, & IBRD / The WB. (2023). *Tracking universal health coverage 2023 global monitoring report* (p. 156). <https://www.who.int/publications/i/item/9789240080379>

Yazbeck, A. S., Soucat, A. L., Tandon, A., Cashin, C., Kutzin, J., Watson, J., Thomson, S., Nguyen, S. N., & Evetovits, T. (2023). Addiction to a bad idea, especially in low- and middle-income countries: Contributory health insurance. *Social Science & Medicine*, *320*, 115168. <https://doi.org/10.1016/j.socscimed.2022.115168>
